# Fractionation of parietal function in bistable perception probed with concurrent TMS-EEG

**DOI:** 10.1038/sdata.2016.65

**Published:** 2016-08-16

**Authors:** Georg Schauer, Acer Chang, David Schwartzman, Charlotte L. Rae, Heather Iriye, Anil K. Seth, Ryota Kanai

**Affiliations:** 1 Sackler Centre for Consciousness Science, University of Sussex, Brighton BN1 9RH, UK; 2 Centre for Integrative Neuroscience, University of Tübingen, Tübingen 72076, Germany; 3 Clinical Imaging Sciences Centre, Brighton and Sussex Medical School, Brighton BN1 9RR, UK; 4 ARAYA Brain Imaging, Tokyo 102-0093, Japan

**Keywords:** Magnetic resonance imaging, Electroencephalography - EEG, Transcranial magnetic stimulation, Attention, Consciousness

## Abstract

When visual input has conflicting interpretations, conscious perception can alternate spontaneously between these possible interpretations. This is called bistable perception. Previous neuroimaging studies have indicated the involvement of two right parietal areas in resolving perceptual ambiguity (ant-SPLr and post-SPLr). Transcranial magnetic stimulation (TMS) studies that selectively interfered with the normal function of these regions suggest that they play opposing roles in this type of perceptual switch. In the present study, we investigated this fractionation of parietal function by use of combined TMS with electroencephalography (EEG). Specifically, while participants viewed either a bistable stimulus, a replay stimulus, or resting-state fixation, we applied single pulse TMS to either location independently while simultaneously recording EEG. Combined with participant’s individual structural magnetic resonance imaging (MRI) scans, this dataset allows for complex analyses of the effect of TMS on neural time series data, which may further elucidate the causal role of the parietal cortex in ambiguous perception.

## Background and Summary

When a single image has two mutually exclusive interpretations, awareness thereof fluctuates over time. This is called bistable perception. The functional role of the right parietal cortex in facilitating switches between these interpretations has been subject of several neuroimaging studies^1–10^. Since conventional functional magnetic resonance imaging (fMRI) is agnostic towards the causal role of brain activity, several authors made use of transcranial magnetic stimulation (TMS) to inhibit cortical function at sites in the parietal cortex with mixed findings^4,5,11–13^. For instance, Carmel and colleagues^13^ as well as Kanai and colleagues^12^ applied inhibitory TMS to a right parietal regions (ant-SPLr), activity of which is associated with bistable percept switches^1^, and found a shortening of bistable dominance durations. Conversely, Kanai and colleagues^11^ also used inhibitory TMS on a region 3 cm more posterior (post-SPLr), cortical thickness and gray matter volume of which correlated with bistable percept durations, and found a lengthening of dominance durations. Employing a predictive coding framework^14–16^, Kanai and colleagues speculated that this is due to a functional fractionation between these two parietal sites, where ant-SPLr is involved in processing top-down predictions, while post-SPLr is involved in processing perceptual error signals^12^. Fluctuating activity patterns in the fronto-parietal network, including specifically ant-SPLr and post-SPLr, could predict the temporal dynamics of bistable perception^9^, as could their fMRI connectivity coupling^17^ and resting state connectivity^18^. These results strengthen the hypothesis that ant-SPLr and post-SPLr have distinct roles in resolving perceptual ambiguity, and that this fractionation may be routed in their functional connectivity.

The purpose of the present dataset is to provide further, rich neuroscientific data to investigate the functional fractionation between ant-SPLr and post-SPLr in bistability, and to shed light on how the dynamic interactions between ant-SPLr and post-SPLr shape bistable perception. In order to scrutinise the effect of parietal neurostimulation on bistability, we chose to combine TMS to these two parietal sites with electroencephalography (EEG), which enables the direct examination of the neural effects of TMS pulses. Crucially, the technique probes the brain’s response to TMS contingent on its current functional state. We applied single pulse TMS (spTMS) to either ant-SPLr or post-SPLr, while participants viewed either a bistable structure from motion rotating sphere, a disambiguated sphere as replay stimulus or during resting-state fixation. Simultaneously we recorded EEG. TMS localisation was performed on the basis of each participant’s individual structural MRI scan. The resulting dataset comprises psychophysical measurements on the temporal dynamics of bistable perception, T1 weighted structural MRI scans as well as epoched multichannel EEG data recorded during the application of spTMS. Next to the analysis of TMS-evoked potentials, the richness of the dataset also allows for more novel and advanced analysis techniques. For instance, combined TMS-EEG has recently been used to distinguish conscious states in humans, such as sleep stages, and aided in the diagnosis of various neurological disorders of consciousness, using measures of complexity in the TMS-evoked EEG response^19–21^. Also, the availability of T1-weighted structural MRI scans facilitates source modelling approaches as well as addressing the question if the TMS effect can be predicted by structural features of the brain. Another application of the data are long range connectivity analyses or investigations of Granger-causality^22,23^. As such, the present dataset can be used to address both methodological questions about the effect of TMS on brain activity, as well as questions concerning the causal role of the parietal cortex in bistable perception.

## Methods

### Participants

18 healthy volunteers with normal or corrected to normal vision were recruited. One participant was not able to see one of the stimulus conditions due to a problem with depth perception, while another participant’s EEG data were unusable. Both were excluded from the study after testing. Yet another participant’s MRI scan displayed an anatomically abnormal 3 mm wide cavity in his right-frontal white matter, which was deemed clinically irrelevant by the attending neuroradiologist and the participant was subsequently tested. (final sample: *N*=16, age 21–30 yrs, mean 24.94 yrs ±3.26 s.d.; 10 female, 13 right handed). Written informed consent was acquired from all participants prior to the study, which was approved by the university’s local ethics committee. Following consent, participants were screened to ensure that they met health criteria for inclusion in a single pulse TMS study.

### Visual stimuli and apparatus

**Figure 1:**
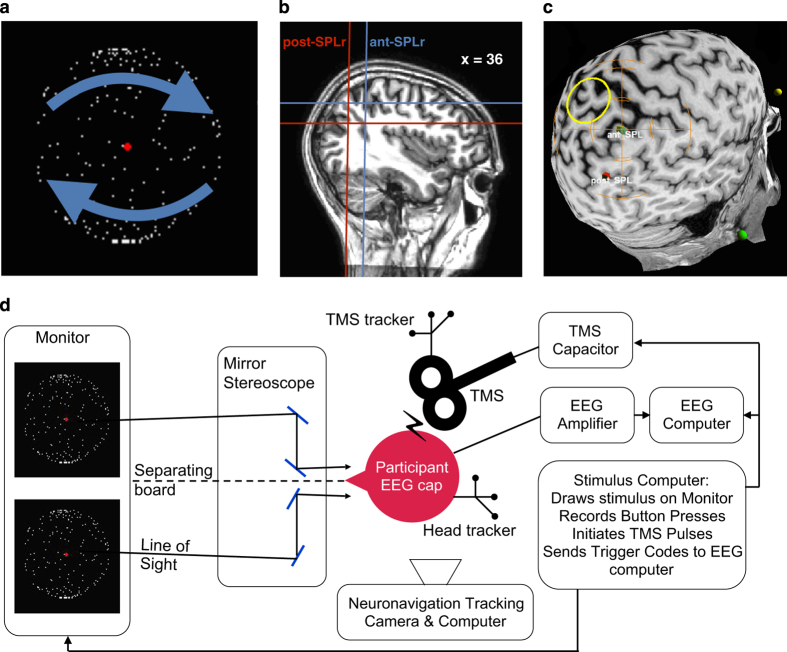
Experimental design. (**a**) Ambiguous rotating sphere. Arrows indicate the two possible movement interpretations of the sphere. (**b**) TMS sites. Ant-SPLr and post-SPLr on the T1 MRI scan of a representative participant. Approximate×coordinate in MNI space for visualisation purposes. (**c**) Neuronavigation. Ant-SPLr and post-SPLr as TMS neuronavigation targets on the cortical surface. Orange cross indicates the current coil position. (**d**) Bird-eye view on experimental setup.

Three stimuli were used in the experiment. 1) The bistable perception stimulus was an ambiguous structure from motion rotating sphere. Dots moved horizontally back and forth within the boundary of a circle in a coherent fashion. All dots took the same amount of time to move from one end to the other, so that dots closer to the vertical midpoint of the stimulus moved faster. This created the illusion of a rotating sphere, which can be seen as either rotating to the left or to the right. The dots took 3 s to move once to either side and back to their starting position. The sphere had a central red fixation dot and was 2.7 angular degrees in diameter (Fig. 1a). 2) As control replay stimulus, we presented participants with a rotating sphere identical to the bistable stimulus, but disambiguated by a depth percept. To create this 3D illusion, participants were presented with two images next to each other on a screen, which were then reflected by a mirror stereoscope (Geoscope, Stereo Aids, Australia) so that both images were positioned in the same location in the visual field of the participant on both eyes (Fig. 1d). The mirrors were carefully adjusted for each participant to achieve fusion of the fixation dot. One of the spheres was presented as having moved just ahead of the other, mimicking the slight binocular disparity humans experience when observing 3D objects from two viewpoints / separate eyes. Given this depth percept, the sphere appears no longer ambiguous. Instead, its motion direction corresponds to whether the right or left sphere is ‘ahead’ of the other. The duration for which the replay sphere would turn either direction were sampled from a gamma distribution obtained from a baseline recording of participants percept durations of the bistable stimulus (see experimental design section). 3) Central fixation consisted of the red dot without a sphere. For the bistable stimulus and resting state fixation, we also used the stereoscope, but simply presented identical spheres / dots to each eye so that no depth was perceived.

Stimuli were presented through the mirror stereoscope at a distance of 70 cm from the participant. The total path travelled by light between stimulus presentation and eye was 75 cm. A black separating board in the middle of the setup as well as around the space between monitor and stereoscope prevented participants from seeing anything apart from the intended image (Fig. 1d). All stimuli were displayed on a 19 inch Lacie CRT monitor (120 Hz refreshing rate at 800×600 pixel resolution; Lacie, Paris, France) controlled by a Dell Precision PC on Windows XP, using the Psychtoolbox package for MATLAB R2014b. There was no natural light contamination nor room lighting. A chin rest was used to minimise head movements.

### Experimental design

In a pre-experiment, participants were seated inside an electromagnetically shielded experiment room, asked to put their chin onto the chin rest and instructed to fixate on a red fixation dot that was stereoscopically presented. The stereoscope was adjusted until a binocular match was achieved and participants reported only perceiving one single fixation dot. Following a button press, participants viewed the ambiguous rotating sphere for 5 runs of 120 s. Participants indicated their current percept (sphere moving left or right), by continuously pressing an arrow key corresponding to that rotation direction. All participants used their right hand for button presses. Bistable dominance durations were calculated from those presses and served as behavioural baseline. Next, participants were made familiar with stimulus 2, the disambiguated 3D sphere, to ensure that the disambiguation was visible. Since bistable percept durations follow the gamma distribution^24^, we used individual gamma shape and scale parameters obtained during the baseline recording of participants viewing the bistable stimulus for each participant to model a gamma distribution from whence we sampled dominance durations to be used for the replay stimulus. The pre-experiment lasted about 15 min.

The main experiment was comprised of six experimental conditions in a 3×2 design: While viewing either stimulus 1, 2 or 3, either of two parietal locations was stimulated (ant-SPLr or post-SPLr, see TMS protocol and neuronavigation section). Each condition appeared three times, hence there were 18 trials in the experiment. Each trial lasted 120 s and included 40 TMS pulses. During all trials, one experimenter stood next to the participant and manually applied the pulses with live feedback from the neuronavigation system for maximum spatial accuracy, while another experimenter monitored the EEG recording for noise and any technical problems. In trials containing stimulus 1 or 2, participants indicated their current percept with continuous button presses. In trials containing stimulus 3, participants were asked to fixate on the centrally presented fixation dot without any further task. There were short breaks between all trials, and two larger breaks of 10 min after trial 6 and trial 12. This was done to ensure that participants could rest, recover from TMS, report any problems, and for individual EEG electrodes to be adjusted if necessary. The main experiment lasted about 60 min and comprised a total of 720 TMS pulses.

### Structural MRI scan acquisition

The MRI scans of all but three participants were acquired on a 1.5T Siemens Avanto MRI scanner at the Clinical Imaging Science Centre (CISC), Brighton and Sussex Medical School. For those participants, a T1-weighted MPRAGE sequence (TR=2730 ms, TE=3.57 ms, FOV=240×256×192 mm, voxel size=1×1×1 mm, matrix 256×256, Flip angle 7°, 192 sagittal plane slices, acquisition time 5 min 58 s) was used to acquire structural MR images. The remaining participants (ID numbers 74, 75 and 81) already had a T1-weighted structural MRI scan from a previous study not acquired at the CISC. We therefore advise to take into account possible differences in the scanning sequence for participants not scanned on site (voxel size 1×1×1 mm for all three scans, ID 74 & 81: FOV=256×256×192 mm, ID 75: FOV=256×256×170 mm).

### TMS protocol and neuronavigation

There are individual differences in the TMS intensity required to evoke a measurable neural effect. To control for this variability, every participant was stimulated at a separate stimulation intensity, namely 90% of their resting motor threshold (RMT). To determine the RMT, participants were asked to relax their right hand with their lower arm resting on an armrest. At initially 65% maximal stimulation output, single pulses (frequency<0.3 Hz) were delivered 2 cm anterior to left hemispheric central midline above the ear at a 45° coil angle relative to the ground while the coil shaft pointed directly downward. The coil was moved between pulses until a motor response in the contralateral hand was observable. Staying at that position with the coil, which marks the right hand representation of the primary motor cortex, stimulation intensity was adjusted until the pulses were just able to reliably evoke a motor response (6 out of 10 times). The mean RMT was 76.13% maximum stimulator output (±5.78 s.d.).

During experimental trials, a single TMS pulse was delivered once every 3 s. These pulses were applied to either the post-SPLr (MNI: x=38, y=−64, z=32), or the ant-SPLr (MNI: x=36, y=−45, z=51) (Fig. 1b). TMS pulses were delivered using a Magstim Rapid 2 figure of 8 air-cooled coil (70 mm coil width, maximum field strength=2.2T, Magstim Ltd, Whitland, United Kingdom). Participants did not consume alcohol in the 24 h prior to each session and were well rested (both to avoid risk of a lowered seizure threshold^25^). Any valuables or metallic accessories, jewellery, piercing or electronic devices were removed before stimulation.

The two parietal locations were localised on the basis of observers' anatomical MRI scans using the neuronavigation system Visor2 (ANT, Enschede, the Netherlands). The standard MNI coordinate system was fitted onto participant’s T1-weighted scans and our regions of interest located in FSL 5.0.8 (Oxford, United Kingdom). These were then transformed to correspond to the scanner anatomical coordinates for each participant, which were subsequently fed into the neuronavigation system. Next to the parietal locations, also the participants’ nasion and the notches posterior to the tragi on both ears were marked on the MRI scans. An infrared tracking system was affixed to the participant which tracked their head motion in space using a Polaris infra-red camera (Northern Digital, Waterloo, Canada). Next, the nasion and tragi were located on the participant and their location marked in relation to the head-tracker. This was used to extrapolate the location of our target region of interest within the room. Another set of trackers was used to trace the position of the TMS coil (Fig. 1d). Knowing both the coil’s as well as the ROI’s location in space, we minimised the distance between the centre-point of the TMS coil and parietal target location. The coil was held manually by the experimenter with its handle pointed directly downwards. The distance between actual coil location and its optimal positioning was kept less than 1.5 mm at all times (Fig. 1c).

### EEG recording and preprocessing

EEG was recorded during the entirety of the main experiment. Prior to the experiment, participants were asked to clear and clean the skin above and below their right eye, their temples and mastoids. At these locations, two vertical electrooculogram electrodes for detecting blinks, two horizontal electrooculogram electrodes to monitor saccades, and two mastoid reference electrodes were attached. Next, head-circumference was measured and an appropriately sized EEG cap put on the participant, ensuring that the Cz electrode lay directly above the vertex. The 64 channel Waveguard EEG electrode layout was based on the extended international 10–20 system, and was connected to a TMS compatible Refa-64 amplifier (Refa 8, ANT, Enschede, The Netherlands). This was followed by the injection of electro-conductive gel into the electrode spaces while simultaneously monitoring impedances, which were kept below 5 kΩ for all electrodes. The live recording was digitised at a sampling rate of 2048 Hz. Participants were asked during each block to be still and to avoid if possible any eye blinks, eye movements, muscle contractions, stretching or any sort of movement that would cause EEG artefacts.

### Code availability

The experimental scripts that displayed the visual stimuli and recorded the behavioural data, those that preprocessed the EEG data as well as those analysing the behavioural data are all available from the authors upon request. All scripts were written in Matlab R2014b using Psychtoolbox3 (ref. 26) (http://psychtoolbox.org), EEGLab^27^ (http://sccn.ucsd.edu/eeglab) and ERPLab^28^ (http://erpinfo.org/erplab) packages, or in R using RStudio 0.98.501.

## Data Records

Participants were assigned a random number between 10 and 99, which was used as designator in all data files. The main experiment was comprised of six experimental conditions (3 stimuli×2 TMS sites). Throughout all data files also the following condition numbers were used:

Condition 1=bistable percept—ant-SPLr

Condition 2=replay stimulus—ant-SPLr

Condition 3=resting state fixation—ant-SPLr

Condition 4=bistable percept—post-SPLr

Condition 5=replay stimulus—post-SPLr

Condition 6=resting state fixation—post-SPLr

### Behavioural data

All behavioural data is available at Dryad repository (Data Citation 1). Each participant’s data are saved in a separate file, e.g., ‘psychophysics_participant_[participant_numer].mat’. The file is a Matlab structure array containing five fields: button presses for the pre-experiment are saved in ‘pre_button’, those for the main experiment in ‘main_button’. Both are a matrix in which each column is a separate trial. Each row is a frame in the presentation of a stimulus. At a refresh rate of 120 Hz this means that 120 rows comprise a second of experimentation. For each frame, a value of 1 indicates a right button press, a value of −1 a left button press and a value of 0 that no button was pressed. ‘pre_gamma’ contains the gamma shape and scale parameter resulting from the pre-experiment. Dominance durations for the main experiment’s replay stimulus were sampled from this gamma distribution. ‘main_conditions’ is a vector containing the order in which conditions appeared through the trials. Lastly, ‘main_output’ summarises statistics computed from the experiment, each row is one of the five experimental runs. The columns are trial number, median dominance for the sphere turning left, median dominance for the sphere turning right, median dominance for all percepts, number of percept switches and total mixed percept durations, as well as gamma shape and scale parameters for each trial’s dominance durations.

### EEG recordings

All EEG data is available at Dryad repository (Data Citation 1). The data are baseline-corrected epochs for time intervals from −510 ms (baseline) to plus 2010 ms around the TMS pulses in each condition. Each participant's data is saved in a separate file, e.g., ‘[participant_number]_EEG_data.mat’. The file is a Matlab structure array containing eight fields: six matrices containing neural time series data for each condition, a time stamp vector and information pertaining to the EEG channels. Data was extracted from EEGLab's EEG structure array^27^ in the following way:

data.cond1=EEG_bin{1}.data;

data.cond2=EEG_bin{2}.data;

data.cond3=EEG_bin{3}.data;

data.cond4=EEG_bin{4}.data;

data.cond5=EEG_bin{5}.data;

data.cond6=EEG_bin{6}.data;

data.time_stamp=EEG.times;

data.channel_info=EEG.chanlocs;

data.cond

The number behind data.cond corresponds to the condition number introduced above. The neural time series data is stored in a separate matrix for each condition. The matrix has the dimensions channel×time×trial, e.g., [66, 5160, 120].

66: There are 66 channels. 5160: Each epoch is 2.52 s long. At a sampling rate of 2048 Hz, this gives us a total of 5160 time points. 120: We originally recorded 120 trials for each condition for each participant, however, due to artefact rejection, this number is smaller in the dataset, for instance for participant 17, condition 1, there are 110 trials.

data.time_stamp

Each participant's datafile also includes a vector called time_stamp. It contains the sampling moments related to the timing of the TMS pulse. For instance, time 1 in the data matrix corresponds to −509.77 ms prior to TMS. The TMS pulse occurs at time point 1045, and data is set to the linear function mentioned earlier between time points 993 (−26 ms) and 1066 (+11 ms).

data.channel_info

This contains some info about the individual channels 1 to 66, such as label and location coordinates, taken directly from the standard EEGLab structure corresponding to the cap setup we used.

### Structural MRI scans

All structural MRI data is available at the Dryad repository (Data Citation 1). There are 16 files in the repository, one scan for each participant in NIFTI format. The filename is equivalent to the participant number.

## Technical Validation

### Behavioural data and TMS carry-over effects

One assumption of this study was that spTMS can be used to probe the brain’s state by analysing the effect of single TMS pulses on scalp electrodes. This in turn relied on the assumption that the probed brain state was similar across all trials in any one condition and that spTMS did not have carry-over effects between the parietal test sites. Such effect would have been likely, if we had used inhibitory repetitive TMS or theta burst stimulation. In previous studies, use of such inhibitory TMS protocols designed to produce ‘virtual lesions’ has led to chances in dominance durations during TMS^4,5^ as well as after^11–13^. By contrast, in our study we used a single pulse TMS protocol, which is meant to probe the brain’s state at single snapshots in time. However, it is not intended to systematically inhibit the function of the targeted areas. Consequently we were not expecting behavioural effects. Instead, behavioural effects would have been evidence for a perturbation of cortical function that we had not intended. Since inhibitory TMS is known to have aftereffects following stimulation^25^, we could in that case not be sure that TMS effects from one parietal test site did not affect the recordings during stimulation of the other.

**Figure 2:**
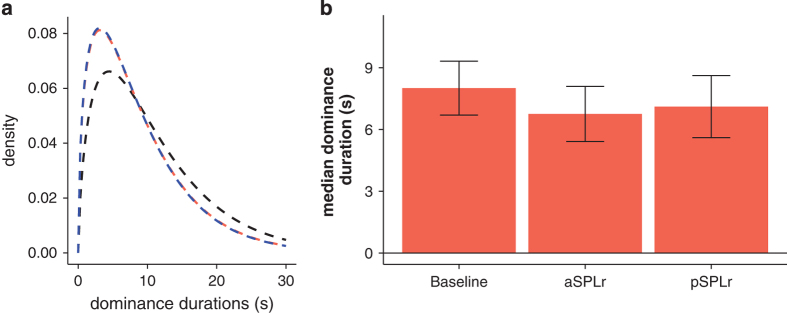
Behavioural results. (**a**) Dominance duration distributions. Gamma distributions from averaged shape and scale parameters across participant’s rivalry recordings from baseline (black), during TMS to ant-SPLr (blue) and post-SPLr (red). (**b**) Dominance duration changes. Median dominance durations during baseline and parietal TMS recordings. Error bars are±1 s.e.m.

One way to test for such carry-over effects is to examine whether bistable perception dominance durations are different between stimulation sites compared to baseline. Two participants’ button report included missing data (17 and 36) and were not entered into this control analysis. Median dominance durations were 8.01 s (±4.91 s.d.) for baseline, 6.75 s (±5.02 s.d.) during ant-SPLr stimulation and 7.11 s (±5.64 s.d.) during post-SPLr stimulation (Fig. 2). We entered the average of the median dominance durations for the three baseline recordings, the three ambiguous ant-SPLr and ambiguous post-SPLr recordings into a one-way repeated measures ANOVA and found no significant main effect of whether of whether the recording was baseline, from ant-SPLr stimulation or post-SPLr stimulation (F(1,13)=0.46, *P*=0.64). We calculated a Bayes factor of 0.2 in favour of an experimental effect, which is considered substantial evidence for the null hypothesis^29^. This means that spTMS to one location did not change dominance durations in comparison to the pre-experiment baseline or the other. It is therefore less likely that TMS carry over effects are an explanation of any experimental effect.

### MRI data and voxel-based morphometry

Based on the previous finding of a significant correlation between percept dominance durations and gray matter volume using small volume correction over both the post-SPLr^11^ as well as ant-SPLr^12^, we conducted an identical voxel-based morphometry analysis over these sites using the T1-weighted structural MRI images of this study’s participants. First, gray and white matter were segmented using the automated segmentation algorithms of the Matlab package SPM8 (http://www.fil.ion.ucl.ac.uk/spm), which was followed by inter-subject registration of only the gray matter images using the ‘diffeomorphic anatomical registration through exponentiated lie algebra’ (DARTEL) function. The resulting images were smoothed with a Gaussian kernel of FWHM=8 mm and then mapped onto the Montreal Neurological Institute (MNI) template. In a regression analysis over the whole brain, participants’ gender and sex were included in the design matrix as covariates of no interest, and a family-wise error corrected alpha level of 0.05 was used to identify voxels which showed a significant correlation between percept durations and gray matter volume. Next, given the strong theoretical support for correlations at specific regions of interest (ROIs), specifically the ant-SPLr and post-SPLr, another regression analysis was carried out predicting dominance durations from gray matter volume over only ant-SPLr and post-SPLr using small volume correction with a sphere radius of 15 mm, centred on co-ordinates (x, y, z) for ant-SPLr and (x, y, z) for post-SPLr.

No positive or negative correlation between percept dominance durations and gray matter volume reached statistical significance following correction for multiple comparisons across the whole brain. Even without correction and using a liberal significance threshold of *P*<0.05, neither ant-SPLr nor post-SPLr showed a significant correlation. Furthermore, analysis of the two ROIs with small volume correction revealed no significant correlations. Hence, the VBM findings by Kanai and colleagues^11,12^ could not be replicated. However, at a sample size of 16, this is not surprising and likely due to a lack of power, since VBM studies usually require substantial larger sample sizes to yield significant results.

### EEG data diagnostics and TMS artefact correction

TMS pulses lead to electrical interference that is several orders of magnitude larger than cognitively evoked EEG signals. This is especially problematic since the recording system requires time to recover from any TMS artefact. Newer systems are able to keep this time to a minimum, however, even in our recordings, the data from TMS onset to about 10 ms after were unusable, and up to about 30 ms after the TMS pulse were highly distorted. To overcome this, the first step of data processing concerned the correction of these TMS artefacts. We used the automated correction algorithm of the Asalab TMS-EEG recording software (ANT, Enschede, The Netherlands), which uses a backward infinite impulse response filter on a fixed interval (−25 ms to 10 ms around TMS pulse onset) and includes a low-pass filter of 100 Hz on that time interval to capture the signal energy of the TMS artefact, which peaks at 300 Hz. Also within that time interval, a least-squares regression equation was used to reconstruct the signal around the artefact on the assumption that the artefact voltage follows the parameters of exponential functions.

Following artefact correction, EEG data were processed offline with ERPLAB^28^, a plugin for the EEGLAB toolbox in Matlab R2013a (Mathworks, USA). Epochs were extracted for time intervals from −510 ms (baseline) to plus 2010 ms around the TMS pulses in each condition and were baseline corrected. The result of this procedure is an EEG signal that appears clean and artefact clear, except for the time interval −25 ms to 10 ms around TMS pulse, which on some trials includes noise several orders of magnitude greater than the signal. Since this time interval is both theoretically and practically uninterpretable, we replaced the signal within that window with a linear equation connecting the voltage at −26 ms with the voltage at +11 ms. Consequently, the signal appears as if the time series data around the TMS pulse had been replaced with a straight line. Trials with peak to peak voltage differences of more than 200 μv within a moving 200 ms time window representing ocular artefacts were removed. Similarly, trials with peak to peak voltage differences of more than 500 μv within a moving 1000 ms time window representing electromyogram bursts and skin potentials were also taken out of the final data. Thereafter, the data were visually inspected to reject any single trials contaminated by muscle and ocular artefacts not excluded by the above algorithm.

**Figure 3:**
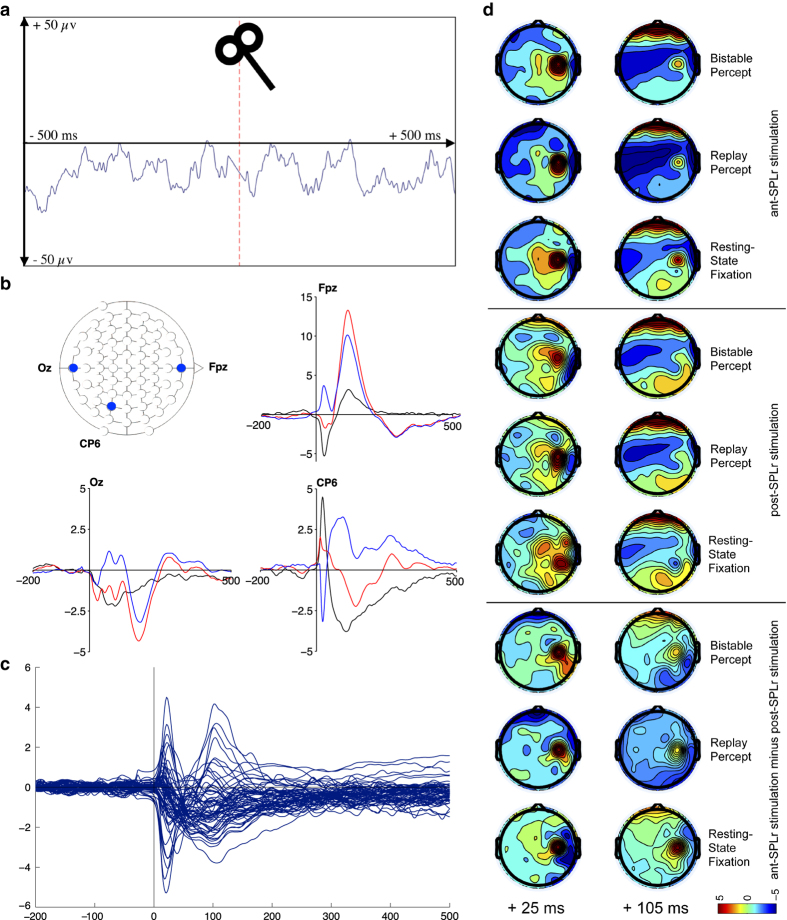
TMS effect on the EEG recording. (**a**) TMS artefact correction. EEG recording of the Pz electrode around a TMS pulse after TMS artefact correction. Single trial in a representative participant. Red line indicates the moment of TMS stimulation. (**b**) TMS-evoked potentials. Baseline-corrected grand average TMS-evoked potentials for electrodes Oz, CP6 and Fpz for the bistable percept stimulus when ant-SPLr is stimulated (red), post-SPLr is stimulated (blue) as well as the difference wave between the two (black). x=time in ms. y=voltage in μv. Electrodes within the 10–20 system top-left. (**c**) Butterly plot. Difference wave butterfly plot of all electrodes apart from C4, CP4, eye and mastoid electrodes contrasting ant-SPLr minus post-SPLr for bistable perception. x=time in ms. y=voltage in μv. (**d**) Scalp maps. Topographic scalp maps of absolute voltage from −5 to +5 μv at 25 and 105 ms post TMS divided by the six experimental conditions (stimulus 1, 2 or 3×TMS site ant-SPLr or post-SPLr) as well as for the three stimuli when post-SPLr amplitudes are subtracted from ant-SPLr amplitudes, prior to any spatial filtering. Colour scale depicts voltage in μv.

Validation of the success of the TMS artefact correction procedure was performed visually. Figure 3a shows one second worth of the Pz electrode EEG recording around a TMS pulse in a representative participant after the correction procedure and data preprocessing in Matlab. The last usable amplitude at −26 ms prior to TMS pulse onset is visibly connected with a mock signal derived from a linear equation with the first usable amplitude post TMS at +11 ms. The resulting waveform shows no abnormalities resulting from TMS that would preclude analysis using standard EEG techniques.

Next, we checked grand-averages over three representative electrodes, Fpz, Oz, and CP6 (Fig. 3b). Of these three, CP6 would be expected to be most problematic because of its proximity to the TMS stimulation site. Plotted are baseline corrected group-level grand averages for the bistable percept around stimulation of either ant-SPLr, or post-SPLr as well as their difference wave. Visibly, the TMS artefact correction procedure was successful in removing the characteristic waveform distortion resulting from TMS, which is in the order of hundreds of μv. Further examining the difference waves across all electrodes (excluding C4, CP4, eye-reference and mastoid, see usage notes) revealed differences in how TMS pulses are dispersed throughout the brain between these sites. In the bistable percept condition, these differences are most notable at around +25 ms as well as +105 ms post TMS (Fig. 3c). Since these differences between sites cannot be due to auditory and tactile components due to subtraction of the waveforms between the two sites, it stands to reason that they capture the functional fractionation of parietal function in bistability observed by Kanai and colleagues^12^.

Despite the correction of visible TMS-EEG artefacts in individual trials, the worry remains that residual artefacts remain on a group-grand-average level. While the actual electrical interference of the TMS on the EEG signal is corrected, participants consistently experienced muscle twitches and ocular movement resulting from TMS pulses. Examining topographical scalp maps reveals that TMS still markedly disturbs the signal, most notably around the parietal stimulation sites at +25 ms post TMS and over frontal regions +105 ms post TMS. We advise care in choosing analyses that are robust against these remaining signal distortions.

### TMS adverse effects

Participants reported muscle twitches and tingling during spTMS stimulation, consistent with previous reports from TMS studies. Also, two participants reported moderate scalp pain and a separate two reported moderate trouble concentrating. There were no serious incidents resulting from TMS application.

## Usage Notes

There are two limitations to the study’s design that are relevant for usage of the data: 1) Electrodes C4 and CP4 lay immediately underneath the TMS coil during the recording and their data are essentially unusable due to noise, especially in the high frequency range above 30 Hz. There is also substantial noise over the mastoid electrodes. Re-referencing to them for ERP type analyses might result in spurious findings. In the EEG recording for participant 26, noise over either C6 or Fc6 was noticeable, and for participant 80, the P3 signal was essentially unusable. 2) For unknown reasons and in about 20% of trials, there is an abrupt change in voltage at 1500 ms post TMS pulse, as the EEG system reset the zero point of voltage amplitudes recorded. We speculate that this is due to the EEG recorder reaching the maximum absolute voltage it can record and essentially subtracting a set amount from the raw signal to compensate. For this reason, we advise caution in analysing epochs longer than +1500 ms post TMS.

The experimental design focused entirely on recording brain-state-dependent EEG responses to TMS pulses. As such, we were not initially interested in the EEG signature of bistable percept switches in the way other studies have investigated them^30,31^. Therefore, we did not record EEG triggers pertaining to perceptual reversals and have epoched EEG data around TMS pulses regardless of percept dominance. The reason that we did not time TMS pulses to percept switches was the fear that participants’ anticipation of oncoming pulses would introduce a response bias and confound the EEG signal. Given the variability in dominance durations from percept to percept, it would not have been possible to reliably time TMS pulses prior to percept switches either. However, for analyses aiming to examine percept switches rather than TMS pulses, we are happy to provide data un-epoched to TMS pulses as well upon request. Instead of EEG triggers, the behavioural data indexed the current percept at 120 Hz (to an error of ±8.33 ms seconds.). This can be used to create EEG time stamps if needed.

## Additional Information

**How to cite this article:** Schauer, G. *et al.* Fractionation of parietal function in bistable perception probed with concurrent TMS-EEG. *Sci. Data* 3:160065 doi: 10.1038/sdata.2016.65 (2016).

## Supplementary Material
